# Extracorporeal cell therapy of septic shock patients with donor granulocytes: a pilot study

**DOI:** 10.1186/cc10076

**Published:** 2011-03-03

**Authors:** Jens Altrichter, Martin Sauer, Katharina Kaftan, Thomas Birken, Doris Gloger, Martin Gloger, Jörg Henschel, Heiko Hickstein, Ernst Klar, Sebastian Koball, Annette Pertschy, Gabriele Nöldge-Schomburg, Dierk A Vagts, Steffen R Mitzner

**Affiliations:** 1 Department of Medicine, Division of Nephrology, Medical Faculty of the University of Rostock, Ernst-Heydemann-Str. 6, Rostock, D-18057, Germany; 2 Department of Anesthesiology and Intensive Care Medicine, Medical Faculty of the University of Rostock, Schillingallee 35, Rostock, D-18057, Germany; 3 Department of Medicine, Division of Transfusion Medicine, Medical Faculty of the University of Rostock, Ernst-Heydemann-Str. 6, Rostock, D-18057, Germany; 4 Department of Medicine, Intensive Care Unit, Medical Faculty of the University of Rostock, Ernst-Heydemann-Str. 6, Rostock, D-18057, Germany; 5 Department of Surgery, Medical Faculty of the University of Rostock, Schillingallee 35, Rostock, D-18057, Germany

## Abstract

**Introduction:**

Neutrophil granulocytes are the first defense line in bacterial infections. However, granulocytes are also responsible for severe local tissue impairment. In order to use donor granulocytes, but at the same time to avoid local side effects, we developed an extracorporeal immune support system. This first-in-man study investigated whether an extracorporeal plasma treatment with a granulocyte bioreactor is tolerable in patients with septic shock. A further intention was to find suitable efficacy end-points for subsequent controlled trials.

**Methods:**

The trial was conducted as a prospective uncontrolled clinical phase I/II study with 28-day follow-up at three university hospital intensive care units. Ten consecutive patients (five men, five women, mean age 60.3 ± 13.9 standard deviation (SD) years) with septic shock with mean ICU entrance scores of Acute Physiology and Chronic Health Evaluation (APACHE) II of 29.9 ± 7.2 and of Simplified Acute Physiology Score (SAPS) II of 66.2 ± 19.5 were treated twice within 72 hours for a mean of 342 ± 64 minutes/treatment with an extracorporeal bioreactor containing 1.41 ± 0.43 × 10E10 granulocytes from healthy donors. On average, 9.8 ± 2.3 liters separated plasma were treated by the therapeutic donor cells. Patients were followed up for 28 days.

**Results:**

Tolerance and technical safety during treatment, single organ functions pre/post treatment, and hospital survival were monitored. The extracorporeal treatments were well tolerated. During the treatments, the bacterial endotoxin concentration showed significant reduction. Furthermore, noradrenaline dosage could be significantly reduced while mean arterial pressure was stable. Also, C-reactive protein, procalcitonin, and human leukocyte antigen DR (HLA-DR) showed significant improvement. Four patients died in the hospital on days 6, 9, 18 and 40. Six patients could be discharged.

**Conclusions:**

The extracorporeal treatment with donor granulocytes appeared to be well tolerated and showed promising efficacy results, encouraging further studies.

**Trial registration:**

ClinicalTrials.gov Identifier: NCT00818597

## Introduction

Despite tremendous advances in critical care medicine, sepsis is still a leading cause of morbidity and mortality in non-coronary ICUs. In the USA, approximately 215,000 patients die each year as a consequence of sepsis [1]. The often unsuccessful efforts to rescue septic patients in ICU are extremely expensive and costs are approaching US $17 billion annually in the United States [1].

The underlying deregulated immune mechanisms that lead to the development of sepsis are highly complex and involve both overshooting inflammatory responses of the innate immune system and the lack of adequate anti-microbial immune responses both by the innate and adaptive arm of immunity. In particular, neutrophils, the prototype of non-specific early anti-microbial effector cells, may lead to collateral damages such as disruption of endothelial integrity and impairment of microcirculation within organs, for example, by overproduction of proteases and oxygen radicals [2-4]. On the other hand, the physiological effector functions of neutrophils are believed to be essential to control the microbial load. Moreover, functional impairment of neutrophils and other immune cells has been shown to be associated with increased mortality in advanced stages of sepsis and septic shock [5-7].

In the past, efforts to stimulate the innate immune system with granulocyte-colony stimulating factor (G-CSF), granulocyte-macrophage-colony stimulating factor (GM-CSF) or interferon gamma (IFN-gamma) in septic patients failed to decrease mortality rates in septic patients. However, except for neonates, no sufficiently powered studies were performed in this field [8-10]. Likewise, the transfusion of granulocyte preparations (GTx) failed to improve survival in sepsis and neutropenia [11,12]. Nevertheless, there is some indication that steroid- or G-CSF-stimulated high-yield granulocyte-donations might result in better survival in severe infections associated with neutropenia and cancer [12,13].

In order to deploy the beneficial features of neutrophils such as phagocytosis of cellular debris, antigenic material or pathogens, and at the same time to circumvent the possible damaging local effects of systemically transfused neutrophils, a bed-side bioreactor was developed, that uses granulocytes in a strictly extracorporeal mode. This bioreactor consists of a plasma separating device and an extracorporeal circuit containing donor neutrophils. The patient is connected to the extracorporeal circuit for the whole treatment. Plasma from septic patients is perfused through the neutrophil housing and the treated plasma is re-infused online into the patient. The bioreactor-cells are retained in the extracorporeal system and discarded after the treatment.

In *in vitro *studies [14] and in a large animal model for Gram-positive sepsis [15], we were able to show the proof of principle and promising survival data. Therefore, the bioreactor is now being studied in patients with septic shock in order to show tolerability and feasibility of this kind of complex therapy. Furthermore, this pilot trial should give hints for relevant end points to adequately power a subsequent controlled study. This is the first report showing data from a pilot study on ICU on the efficacy and tolerability of a granulocyte bioreactor system.

## Materials and methods

The study was conducted in accordance with the Helsinki Declaration, received ethics approval from the local research ethics committee, and the state authorities were notified according to German pharmaceutical and medical device law. The trial has been registered at ClinicalTrials.gov under reg.-no: NCT00818597. Written informed consent was obtained from all participants or from the patients' representatives if direct consent could not be obtained.

### Patients

During a four-month period all patients of one medical and two surgical intensive care units of a tertiary care university hospital were screened to see if they fulfilled the parameters of severe sepsis and septic shock as defined by international consensus criteria [16]. Definitions of organ dysfunctions were adopted from the "Recombinant Human Activated Protein C Worldwide Evaluation In Severe Sepsis Study" (PROWESS Study) [17] with the difference being that liver failure was not an exclusion criterion in this current study. The exclusion criteria were age under 18 years, hepatitis C, active bleeding and HIV infection. Ten consecutive patients with septic shock were enrolled in the study.

### Procedures

**Figure 1:**
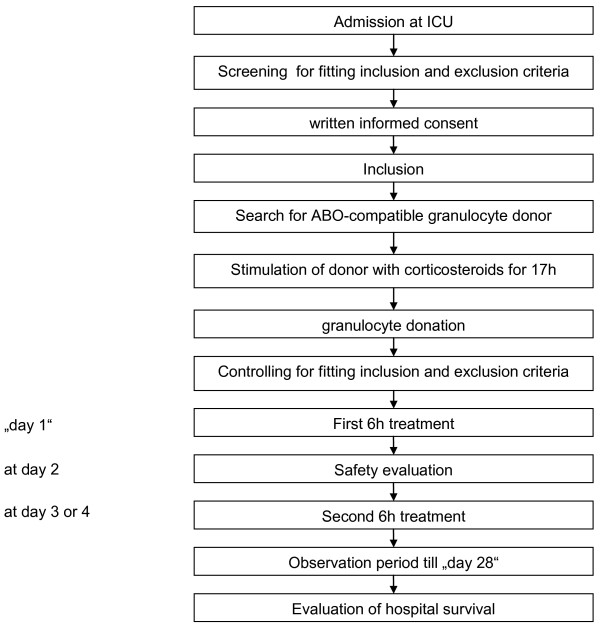
**Schematic view of the study flow**.

**Figure 2:**
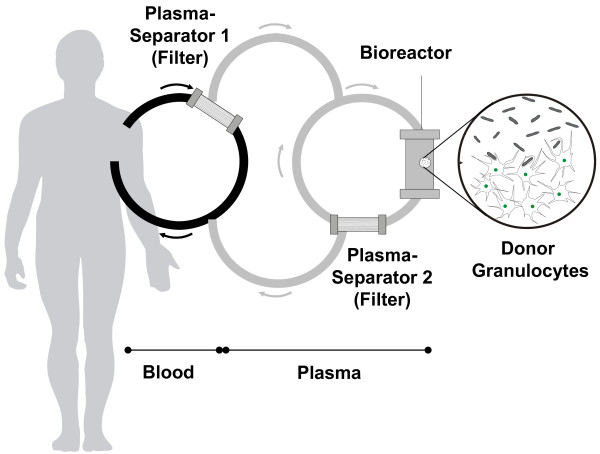
**Schematic drawing of the extracorporeal treatment**. Plasma is separated from blood, transferred to the cell-compartment, and then returned to the patient.

The study flow is depicted in Figure 1. After inclusion of a patient, a healthy blood donor was identified and stimulated with corticosteroids (each 20 mg p.o. methylprednisolone, Sanofi-Aventis Deutschland GmbH, Frankfurt, Germany) 17 h, 12 h and 2 h before donation of an ABO-compatible granulocyte concentrate. Granulocytes were collected by extracorporeal density gradient centrifugation using hydroxyethylstarch (HES 200/0.5 6%, Fresenius Kabi AG, Bad Homburg, Germany) and citrate in a cell separator (COBE Spectra, Gambro BCT, Planegg-Martinsried, Germany) according to standard procedures. Because of the delay due to identification and stimulation of a compatible donor the first treatment of a patient was one day after inclusion in four cases, two days after inclusion in three cases, and three days after inclusion in two cases. Prior to treatment the inclusion criteria were re-confirmed. The whole extracorporeal system was first rinsed and prefilled with hemofiltration solution HF-BIC 35-410 with 4 mM potassium (Fresenius Medical Care, Bad Homburg, Germany). In mean 1.41 ± 0.43 × 10E10 donor granulocytes were delivered in donor plasma and were placed into the bioreactor compartment of the device prior to connection to the patient. An excess of hemofiltration solution during cell filling was discarded; therefore, no additional fluid was infused into the patient. The patients were treated for up to six hours with an extracorporeal method consisting of a plasma separation and plasma perfusion through the cell-compartment containing the donor cells. Blood access was veno-venous via a Shaldon-catheter. Plasma separation was carried out by a dialysis monitor (BM25, Edwards Lifesciences GmbH, Unterschleissheim, Germany) using a 0.5 μm pore-size plasma filter (PF 1000N, Gambro Hospal GmbH, Planegg-Martinsried, Germany). The plasma was infused into the continuously re-circulating donor cell compartment. A schematic view of the extracorporeal treatment device is shown in Figure 2. Plasma reflux to the patient was done through a second PF 1000N plasma filter to withhold the donor cells from being infused into the patient. Total extracorporeal volume was 400 ml. The blood flow rate was 150 to 200 ml/minute with a plasma separation rate of 16.7 to 33.3 ml plasma/minute using the BM 25 monitor. The MARS-Monitor 1 TC (Gambro Rostock GmbH, Rostock, Germany) was used for the re-circulating bioreactor circuit at a rate of 200 ml/minute and to maintain the temperature in the cell compartment at 37°C. Unfractionated heparin (20 IU/kg, Roche, Grenzach-Wyhlen, Germany) was given at the beginning of the extracorporeal treatment followed by a continuous infusion into the circuit. Heparin administration was adjusted to maintain activated clotting time (ACT) between 150 to 200 seconds. Following tolerability assessment of the first treatment, all patients were treated a second time 48 to 72 hours after the first treatment, again for up to 6 hours with granulocytes from the same donor.

### Measurements

We recorded basic demographic information, illness severity (Acute Physiology and Chronic Health Evaluation (APACHE) II, Sequential Organ Failure Assessment (SOFA), Multiple Organ Dysfunction Score (MODS), and Simplified Acute Physiology Score (SAPS) II scores), microbiological results, pre-morbidity, and clinical outcome for the study cohort (see Table 1). Patients were followed up for 28 days and hospital survival. At the days "inclusion", 1 to 8, 10, 12, 14, 21, 28 and before/after an extracorporeal bioreactor-treatment the patients were screened for clinical and immunological data: hemodynamic, inflammation, coagulation, hemolysis, temperature, organ function blood parameters, endotoxin, cytokines, complement (C3, C4), and the number of human leukocyte antigen DR (HLA-DR) molecules per monocyte surface. "Day 1" was defined as the day of the first bioreactor treatment. Viability and functionality of the donor cells were tested at the begin and end of the treatments by trypan blue test, phagocytosis by flow cytometry (Beckman Coulter Immunotech, Krefeld, Germany) with florescence-labeled E. coli and oxyburst both by flow cytometry with dihydrorhodamine 123 as well as in a luminometer (Thermo Labsystems, Waltham, MA, USA) with luminol and lucigenin.

**Table 1:** Patients characteristics, illness severity, premorbidity and clinical outcome for study cohort (*n *= 10).

Patient	Major diagnoses at inclusion	Premorbidity	APACHE II at ICU arrival	SOFA at ICU arrival/at inclusion	SAPS II at ICU arrival/at inclusion	Hospital survival	Inclusion at ICU day	Time between inclusion and first treatment in days
1	Pneumonia, ALI, SS	Alcohol abuse	37	15/16	96/80	Survived	1	2
2	Necrotizing pancreatitis, Pneumonia, SS	Alcohol abuse	27	12/11	61/61	Survived	9	1
3	Pneumonia, ALI, Urogenital infection, SS	Ischemic heart disease, Hydrocephalus, brain-tumor operation	30	12/11	66/58	Died (Day 18)	3	1
4	ALI, SS, Liver failure	Liver cirrhosis, COPD, Diabetes mellitus	37	17/17	72/73	Died (Day 9)	10	1
5	Cardiopulmonary resuscitation, ALI, SS	Alcohol abuse, Encephalopathy, Ischemic heart disease	36	11/13	83/66	Survived	4	1
6	Mediastinitis, SS	Alcohol abuse	27	14/13	70/63	Survived	1	2
7	Hip joint endoprosthesis infection, SS	Diabetes mellitus	21	8/6	35/35	Died (Day 40)	1	3
8	Postoperative shock after ACB-surgery, ARF, SS	Ischemic heart disease, Cardiac failure	38	13/9	74/50	Survived	6	12
9	Renal failure, Kidney infection, SS	Polycystic Kidney and Liver	29	8/12	72/31	Survived	3	2
10	Thoracic infection after sternum resection, ARF, SS	Radio-Necrosis of Sternum after Radio-Chemotherapy due to Breast Cancer	17	8/11	33/70	Died (Day 6)	27	3
Median			29	12/11	70/61		4	2
Mean			29.9	11.8/11.9	66.2/58.7		6.5	2.8

ACB, aortocoronary bypass, ALI, acute lung injury, ARF, acute renal failure, COPD, chronic obstructive pulmonary disease, SS, septic shock

### Statistical analysis

The Statistical Package for the Social Sciences (SPSS, IBM Corporation, Somer, NY, USA) was used to conduct nonparametric analyses using the Friedman-test and Wilcoxon-test. In addition to the evaluation of the raw data, a Last Observation Carried Forward (LOCF) analysis was performed to limit the bias due to the dropout of the three non-survivors during the 28 days observation period. The results are expressed as the mean ± standard deviation (SD). Differences were considered significant at *P *< 0.05.

## Results

### Patients

Ten consecutive patients with septic shock were included in the study. Details concerning diagnoses, age, sex, relevant scores and survival are shown in Table 1. All patients had positive microbial tests with a mean of 4.7 ± 2.6 different microbial species per patient, predominantly candida, coagulase negative staphylococcus, enterococcus and E. coli.

### Observations during the treatments: technical results

During the first treatment performed in this study the heparin use was adjusted to a target ACT of 125 to 150 sec. After about 90 minutes the cell filter clotted and the treatment had to be terminated. Therefore, in all further treatments the heparin dosage was adjusted according to a target ACT of 150 to 200 sec. Except for Patient 6 where treatment #2 had to be terminated after five hours due to increased transmembranal pressure across the cell filter, all other treatments were carried out for six hours. Mean treatment time was 342 ± 64 minutes. Blood flow varied from 150 to 200 ml/minute depending on the patient's quality of blood access. The flow rate in the cell therapy circuit was 200 ml/minute. Plasma flow started with 16.7 ml/minute for the first 15 to 30 minutes and then increased to 33.3 ml/minute. A mean of 9.8 ± 2.5 liters of plasma were treated during each of the 20 treatments. To test whether the donor cells were still functional every two hours, cells from the cell circuit were evaluated for viability and functionality. For the whole treatment the cells showed a viability of more than 90% and unimpaired cellular functions like phagocytosis and oxidative burst.

### Primary endpoints (safety): hemodynamic

During the extracorporeal procedures, no significant drop in mean arterial pressure was observed. All patients were on noradrenaline at the beginning of the first treatment and five of these patients also received it at the start of the second treatment. In 10 of the 20 procedures the noradrenaline dose could be reduced due to an increase in the mean arterial pressure. In five treatments the noradrenaline dose remained unchanged. Only in one case (Patient 4, second treatment) the noradrenaline infusion that had been turned off before the treatment was turned on again during the treatment, however, at a small dose (0.03 μg/kg/minute). Overall the Wilcoxon test showed a significant reduction in the noradrenaline dose (median from 0.06 to 0.035 μg/kg/minute; *P *= 0.016; Table 2) while the mean arterial pressure was stable during the bioreactor-treatment (median before 74, after 80 mmHg; not significant). Systemic vascular resistance index (SVRI) was not monitored in this study.

**Table 2:** Main laboratory parameters before and after the extracorporeal treatments.

Parameter	Unit	Before extracorporeal treatment *n *= 20	After 6 h extracorporeal treatment *n *= 20	*P*-value
Inflammation				
Leukocytes	Gpt/l	12.2 ± 6.6	20.8 ± 12.4	*P *< 0.01
Banded neutrophils	%	73 ± 11	70 ± 10	n.s.
Segmented neutrophils	%	18 ± 11	18 ± 12	n.s.
C-reactive protein	mg/l	190 ± 130	165 ± 119	*P *< 0.01
Procalcitonin	ng/l	10.1 ± 23.4	6.8 ± 14.6	*P *< 0.01
Endotoxin	pg/ml	16.4 ± 7.7	13.5 ± 5.5	*P *< 0.05
Temperature	°C	36.86 ± 0.97	36.73 ± 0.87	n.s.
Hemodynamic				
Noradrenaline	μg/kg/minute	0.10 ± 0.12	0.08 ± 0.10	*P*< 0.05
MAP	mmHg	76.9 ± 12.8	80.2 ± 9.8	n.s.
Pulse	bpm	101 ± 19	101 ± 20	n.s.
Respiration				
PaO2	kPa	13.0 ± 3.1	13.4 ± 4.0	n.s.
FiO2	%	40.8 ± 19.4	40.0 ± 16.3	n.s.
Coagulation				
INR		1.29 ± 0.25	1.44 ± 0.29	*P *< 0.01
aPTT	sec	53.8 ± 50.2	85.8 ± 47.4	*P *< 0.05
Antithrombin III	%	65.6 ± 16.8	58.4 ± 15.3	*P *< 0.01
Fibrinogen	g/l	5.07 ± 2.25	4.62 ± 2.15	*P *< 0.01
D-Dimere	μg/l	752 ± 505	853 ± 450	*P *< 0.05
Platelets	Gpt/l	163 ± 130	169 ± 152	n.s.
Other				
Urea	mmol/l	13.5 ± 7.5	15.0 ± 8.4	*P *< 0.01
Creatinin	μmol/l	129 ± 99	132 ± 108	n.s.
Bilirubin	μmol/l	33.1 ± 44.2	35.4 ± 44.8	n.s.

MAP, Mean arterial pressure; INR, International normalized ratio; aPTT, Activated partial thromboplastin time

### Coagulation disorders

There was no significant change in mean platelet counts during the extracorporeal treatment (Table 2). D-dimers did increase significantly during the extracorporeal treatment from 752 ± 505 μg/l to 853 ± 450 μg/l but returned to 609 ± 381 μ/l within 12 hours. Antithrombin III concentration also changed significantly from 66 ± 17% at the beginning to 58 ± 15% at the end of the treatments, and improved slightly over the following 12 h to 61 ± 15%. Both activated partial thromboplastin time (aPTT) and prothrombin time (as International Normalized Ratio, INR) increased during the treatments due to heparin use but returned to pre-treatment values within 12 h after the extracorporeal circulation. No hemorrhages were observed.

### Hemolysis

No signs of hemolysis were observed. Haptoglobin remained within the normal range and no significant change in lactate dehydrogenase was seen during the treatments.

Moreover, no allergic reactions were recognized.

### Secondary endpoints (safety and efficacy): comparison of projected and observed mortality

Expected in-hospital mortality based on the ICU entrance APACHE II (29.9 ± 7.2) and SAPS II (66.2 ± 19.5) scores were 69.1% and 71.5%, respectively [18-20]. The observed mortality rate was 3 out of 10 within 28 days (on days 6, 9, and 18), and four during hospital stay (Patient 7 died on Day 40). Six patients could be discharged from the hospital in stable condition. No significant differences were seen between the survivors and non-survivors in the time at ICU before inclusion or the time between inclusion and first treatment.

### Organ functions, vital signs and laboratory parameters

The body temperature of the patients was stable during the treatments (Table 2). While creatinine did not show a significant change during the six-hour treatments there were small but significant increases in urea (Table 2), most probably due to interruption of dialysis in patients with renal failure. However, urea decreased again slightly within 12 h post treatment to 14.7 ± 8.4 mmol/l. No difference in PaO2 and FiO2 has been observed between start and end of the extracorporeal treatment (Table 2). Furthermore, no significant changes have been seen in PaO2 or FiO2 between the treatment day and the day after the treatment.

### Inflammation

During the six-hour treatment a dramatic increase in white blood cell (WBC) counts was observed (Table 2). This increase was not due to changes in a particular subset of WBC, the ratio of segmented to banded neutrophils remained unchanged. Furthermore, there was a significant decrease in plasma endotoxin concentration from pre- to post-treatment (Table 2). In 11 of the tested cytokines a significant increase pre vs. post cell-bioreactor was observed (IL-2,-4,-8,-10,-1beta,-12, IP-10, Interferon gamma, Eotaxin, PDGF, RANTES). This resulted in significant increases pre- vs. post-treatment in the patients' plasma in 5 out of these 11 cytokines (IL-8,-10,-1beta, Eotaxin, RANTES) (Table 3). Moreover, there were significant decreases both in CRP as well as in PCT during the treatments (Table 2).

**Table 3:** Changes in cytokine concentrations in patients' bood (left side) and in the extracorporeal circuit (right side).

	Patient				Extracorporeal circuit during treatment			
		
Mediator	Before extracorporeal treatment	After 6 h extracorporeal treatment	%	*P*	Directly before cell compartment	Directly behind cell compartment	%	*P*
IL-2	3.67	11.92	325	n.s.	0.78	1.42	182	**< 0.05**
IL-4	0.87	2.29	263	n.s.	0.09	0.24	268	**<0.001**
IL-6	102.22	313.15	306	n.s.	226.06	299.38	132	n.s.
IL-8	20.39	41.31	203	**<0.05**	31.79	165.15	520	**<0.001**
IL-10	2.57	6.54	254	**<0.01**	3.86	6.02	156	**<0.05**
IL-1 beta	1.21	2.12	175	**<0.05**	0.74	1.11	150	**<0.05**
IL-5	0.42	1.33	315	n.s.	0.39	0.52	135	n.s.
IL-7	3.19	5.19	163	n.s.	2.64	4.14	157	n.s.
IL-12(p70)	2.46	9.65	392	n.s.	0.09	0.45	498	**<0.001**
IL-13	1.68	3.34	199	n.s.	0.85	1.05	124	n.s.
IL-17	0.05	0.59	1185	n.s.	0.04	0.10	274	n.s.
IL-1ra	106.96	208.03	194	n.s.	113.40	134.53	119	n.s.
IL-15	4.19	6.35	151	n.s.	3.20	4.37	136	n.s.
IL-9	1.11	8.15	737	n.s.	0.29	0.78	265	n.s.
IP-10	240.16	561.57	234	n.s.	508.51	749.08	147	**<0.05**
G-CSF	30.84	43.73	142	n.s.	50.87	53.44	105	n.s.
GM-CSF	10.81	50.79	470	n.s.	1.52	3.41	224	n.s.
IFN gamma	50.29	79.07	157	n.s.	14.83	25.26	170	**<0.05**
TNF alpha	0.00	0.00	100	n.s.	0.81	0.19	24	n.s.
MCP-1(MCAF)	130.72	224.46	172	n.s.	299.52	225.67	75	n.s.
MIP-1b	55.93	98.89	177	n.s.	76.92	103.23	134	n.s.
Eotaxin	85.64	216.82	253	**<0.05**	80.23	130.72	163	**<0.01**
FGF basic	1.51	9.47	629	n.s.	0.61	0.00	0	n.s.
PDGF bb	652.01	1145.02	176	n.s.	10.28	62.25	606	**<0.001**
RANTES	137.70	298.64	217	**<0.05**	22.91	141.43	617	**<0.001**
VEGF	168.92	198.68	118	n.s.	1.12	2.45	219	n.s.
MIP-1 alpha	0.39	1.00	253	n.s.	0.34	0.51	148	n.s.

### Results of the 28-day observation period

**Figure 3:**
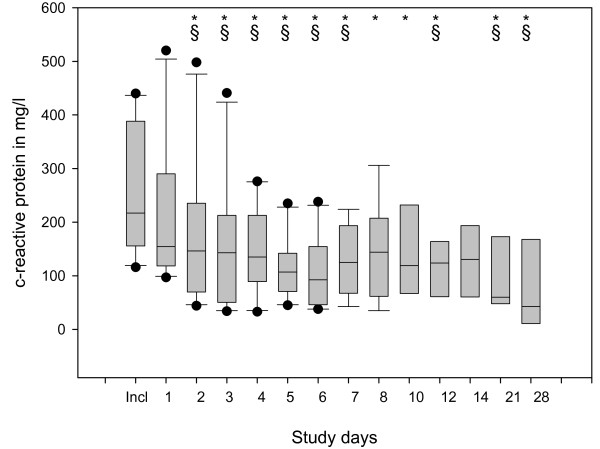
**Box plots of data describing the time course of C-reactive protein**. Significant changes (*P *< 0.05) vs. inclusion day (indicated by *) and vs. Day 1 (§) were observed.

**Figure 4:**
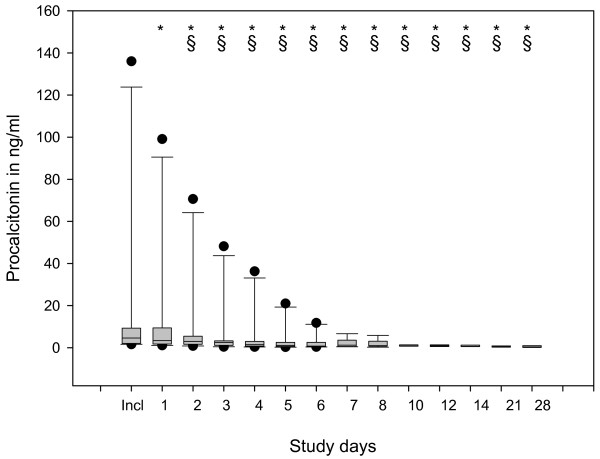
**Box plots of data describing the time course of procalcitonin**. Significant changes (*P *< 0.05) vs. inclusion day (indicated by *) and vs. Day 1 (§) were observed.

**Figure 5:**
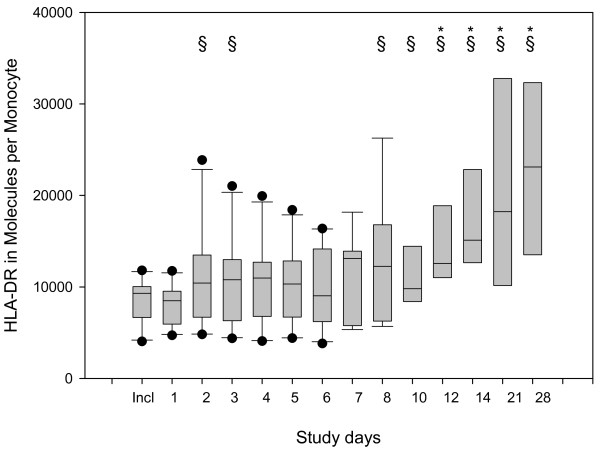
**Box plots of data describing the time course of HLA-DR expression on CD14 positive monocytes**. Significant changes (*P *< 0.05) vs. inclusion day (indicated by *) and vs. Day 1 (§) were observed.

**Figure 6:**
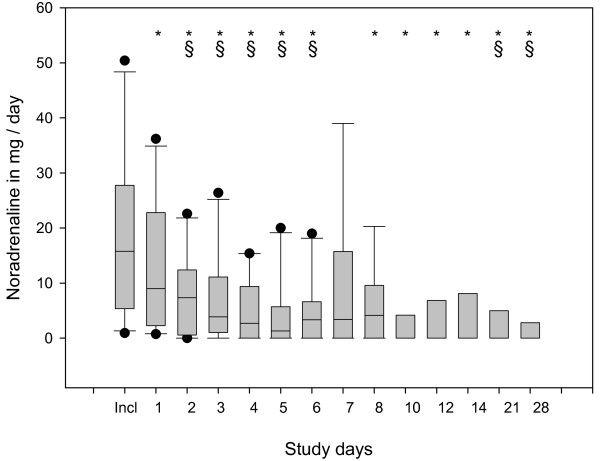
**Box plots of data describing the time course of total daily noradrenaline dosage**. Significant changes (*P *< 0.05) vs. inclusion day (indicated by *) and vs. Day 1 (§) were observed.

The statistical evaluation of the raw data showed improvements in several parameters evaluated during the 28 days of observation. The main findings include: significant reduction in CRP (Figure 3), PCT (Figure 4) and IL-8 (not shown); significant increase in HLA-DR on CD14-positive monocytes (Figure 5); significant increase in platelets and antithrombin III (not shown); significant reduction in noradrenaline use (Figure 6); significant reduction in alanine transaminase, aspartate transaminase and creatinine (not shown); and significant reduction in MODS and SOFA scores (not shown).

Out of these parameters PCT values, Noradrenaline dosage and SOFA score showed improvement already prior to the first treatment and further improved during the observation period.

In order to limit the bias due to the dropout of the non-survivors, an additional LOCF analysis was performed that also showed significant improvements for CRP, PCT, HLA-DR, noradrenaline, and creatinine.

Due to the large inter-individual differences no significant changes in leukocyte counts were seen except directly before and after treatment (see above).

## Discussion

Today's best treatment of sepsis includes early and aggressive antibiotic therapy and effective support for failing organ systems including metabolic stability and maintenance of stable hemodynamic [21]. Immunomodulation has been introduced as an adjunctive therapeutic approach to overcome immune system dysfunction and could show positive impact on survival in some studies [22] but failed in a number of other studies [23,24]. Extracorporeal blood detoxification methods have also been suggested to successfully influence immune imbalances and subsequently clinical course and outcome of multi-organ failure and sepsis [25]. High volume hemofiltration [26], high cut-off hemofiltration [27], high adsorption hemofiltration [28]; coupled plasma filtration adsorption (CPFA) [29]; plasma- or whole blood perfusion through adsorptive columns [30]; and plasma or whole blood exchange have been proposed (for review see [31,32]). Cytokines, for example, can be significantly reduced in the circulation of septic patients by extracorporeal treatments. Techniques capable of removing larger molecules/particles from plasma (that is, high-volume treatments, large-pore filtration, plasmapheresis and adsorption) appear to have a stronger impact on clinical course and outcome than techniques primarily addressing smaller water-soluble molecules [29,33].

Extracorporeal bioreactors were studied in the treatment of various diseases. Acute liver failure [34] and acute renal failure associated with sepsis [35] have been targeted by different cell-based extracorporeal organ support systems using hepatocytes or renal tubular cells. Proper choice of the cell-source turned out to be of central importance [36]. However, the use of immune cells to treat sepsis in an extracorporeal setting has not been reported so far.

Allogeneic blood transfusions have been implicated to increase the risk of nosocomial infections and are independently associated with increased length of stay and mortality in critically ill patients [37]. Leukocytes are thought to trigger this effect and leuko-reduction of blood transfusions was found to result in a decrease of infections and mortality in post-operative intensive care [38]. Therefore, the intravenous transfusion of leukocytes remains under controversial discussion.

In a pig study of Staphylococcus aureus-induced sepsis, the extracorporeal granulocyte-treatment resulted in significant improvement of one-week survival as compared to both the untreated and the sham control. The effect on survival was dependent on the presence of granulocytic HL-60 cells in the bioreactor device. In the sham-bioreactor-treated group no survival benefit was observed [15].

In this current study 10 patients with septic shock were treated. The plasma of the patients had a strong inhibitory effect on the functionality (that is, oxyburst) of myeloid cell lines, indicating a neutrophil function-inhibiting milieu in all patients (data not shown). This is in line with reports in the literature [7].

The extracorporeal cell-treatment was well tolerated both with regard to technical safety of the procedure as well as the biocompatibility of the allogeneic bioreactor-cells. No adverse effects were noted that could be accounted for by the presence of the human phagocytic cells. Specifically, no unwanted effects were observed in the function of the lungs or other organs as were reported following GTx-treatments.

The dosage of anticoagulation needed to be increased following an episode of clotting observed during the first single treatment. For all following treatments a higher target ACT was adopted. After the adaptation, no clotting or increased bleeding episodes were observed.

The hemodynamic situation of the patients improved significantly through the course of the treatment. This is a remarkable finding as other extracorporeal blood treatments such as renal replacement therapies can induce hypotension and other unwanted effects in critically ill patients [39]. There is a correlation between vasopressor load and mortality in septic shock patients [40]. Thus, reduction of vasopressor load might be a valuable parameter for future clinical studies with the bioreactor device.

The increase in leukocyte count after six hours of treatment is one of the results that appear to be a direct effect of the bioreactor perfusion. It most likely is the consequence of a cytokine influx from the bioreactor. However, no clinically unwanted effect of this leukocytosis was observed, neither directly following treatment nor in the following days (that is, no organ dysfunction, especially no notable lung injury). This might be due to the "balanced" cytokine influx with both pro- and anti-inflammatory cytokines (compare Table 3).

The 28-day results indicate stabilization of conditions in seven patients including normalization of the inflammatory situation and reversal of organ failure, resulting in seven 28-day survivors and six hospital survivors. However, no conclusions about survival can be drawn based on this uncontrolled pilot study. Moreover, the favorable clinical course of the majority of patients cannot be linked only to the bioreactor treatments based on the present data. They might just reflect the natural course of the disease and the impact of proper standard intensive care treatment. Future clinical investigations will be needed to address these questions.

The mechanism of action of the device remains incompletely understood at present. The efficient removal of live bacteria by granulocytes was both proven *in vitro *and in the pig-bacteremia model [14,15]. There is already good evidence for removal of bacterial endotoxins as well as interaction on the mediator and cytokine level during this clinical study. This is in line with observations from the pig model [15]. Interestingly, the bioreactor cells released a mixture of pro-inflammatory as well as anti-inflammatory cytokines. The interactions on the cellular and mediator level will be another task to study in future clinical trials.

The present study has several limitations. As an uncontrolled pilot study it does not carry the capacity to answer any questions regarding clinical course or outcome of the patients. Further controlled studies in larger patient cohorts will need to address these questions. Although no severe unwanted effects were observed during the treatments, no final conclusion on the safety can be drawn based on the results from 20 single treatments in 10 patients. The course of biomarkers of inflammation and cytokines needs further investigation as well. The apparent link between fall in CRP and PCT following the bioreactor treatments needs to be separated from the effects induced by standard intensive care including application of antibiotics. The mechanism of cytokine response of the bioreactor needs further elucidation. The observed influx of pro- and anti-inflammatory cytokines into the patient surely is one of the most interesting results of this study. However, it has to be carefully followed in further investigations and its impact on patient's safety should be monitored closely.

At present extracorporeal detoxification methods already play an important role in intensive care therapy of septic multi organ failure, for example, as renal and liver dialysis [41]. A combination of various extracorporeal support approaches appears as an interesting option for future organ support strategies.

## Conclusions

The objective of the current study was to deploy donor granulocytes in patients with septic shock and immune cell-paralysis in a strictly extracorporeal setting and, thereby, prevent potential local side effects in the inflamed tissue.

In summary, the results of the present study mainly indicate three things: a) extracorporeal plasma-treatment with granulocytic cells is well tolerated in critically ill patients with septic shock, b) treatment was associated with significant improvement of the hemodynamic situation of the patients, and c) clinical courses of the patients in this pilot study encourage further clinical studies with this therapeutic approach.

## Key messages

• A bedside-bioreactor with donor granulocytes was clinically tested in 10 patients with septic shock.

• Every patient was treated twice for six hours each. The treatments were tolerated well by the patients.

• The bioreactor cells released a mix of pro- and anti-inflammatory cytokines that had an impact on the cytokine levels in the patient.

• Parameters describing immune cell function (HLA-DR), inflammation status (CRP, PCT, WBC), hemodynamics (vasopressor dosage) of the patients improved during the treatment.

• Labchemical and clinical results encourage further clinical studies.

## Abbreviations

ACT: activated clotting time; APACHE: Acute Physiology and Chronic Health Evaluation (Score); aPTT: activated partial thromboplastin time; CPFA: coupled plasma filtration adsorption; CRP: C-reactive protein; FGF: fibroblast growth factor; G-CSF: granulocyte-colony stimulating factor; GM-CSF: granulocyte-macrophage-colony stimulating factor; GTx: granulocyte transfusion; HLA-DR: human leukocyte antigen DR; IFN: interferon; IL: interleukin; INR: International Normalized Ratio; LOCF: Last Observation Carried Forward; MCP-1: monocyte chemotactic protein-1; MIP-1: macrophage inflammatory protein 1; MODS: Multiple Organ Dysfunction Score; PCT: procalcitonin; PDGF: platelet-derived growth factor; SAPS: Simplified Acute Physiology Score; SD: Standard Deviation; SOFA: Sequential Organ Failure Assessment (Score); SPSS: Statistical Package for the Social Sciences; SVRI: systemic vascular resistance index; TNF: tumor necrosis factor; VEGF: vascular endothelial growth factor.

## Competing interests

JA and SRM have filed patents on the technology of extracorporeal cell perfusion technology, own shares of and work part-time as consultants for Artcline GmbH, a University spin-off that now owns the patent rights. MS works part-time as a consultant for Artcline GmbH. Artcline will pay the article processing charge of this journal. KK, HH, SK, TB, DV, GNS, MG, JH, AP, EK and DG declare that they have no competing interests.

## Authors' contributions

JA did the regulatory work and coordinated the preparation of the manuscript. JA and KK did the data analysis. MS, SRM, HH, SK, TB, DV, GNS, MG, JH, AP and EK were clinical investigators of the study and performed the treatments. DG did the donor screening and the donor granulocyte collections. All authors participated in the design of the study, and read and approved the final manuscript.
